# The function of lncRNA EMX2OS/miR-653-5p and its regulatory mechanism in lung adenocarcinoma

**DOI:** 10.1515/med-2023-0686

**Published:** 2023-04-10

**Authors:** Lina Ma, Lu Zhang, Lin Li, Li Zhao

**Affiliations:** Thoracic Surgical Nursing, Qinhuangdao First Hospital, Qinhuangdao 066000, Hebei, China; Thoracic Surgical Nursing, Qinhuangdao First Hospital, 258 Wenhua Road, Haigang District, Qinhuangdao 066000, Hebei, China

**Keywords:** lung adenocarcinoma, miR-653-5p, lncRNA EMX2OS, prognosis, cellular processes

## Abstract

This study aimed to evaluate the significance of EMX2OS in lung adenocarcinoma (LUAD) prognosis and development and its potential molecular mechanism. Paired tissue samples were collected from 117 LUAD patients. The EMX2OS expression level was detected by PCR and correlated with patients’ clinicopathological features by a series of statistical analyses. The function of EMX2OS in cell proliferation and metastasis was evaluated by CCK8 and Transwell assay. In mechanism, the interaction between EMX2OS and miR-653-5p was assessed by the dual-luciferase reporter assay, and the regulatory effect of miR-653-5p on EMX2OS tumor suppressor role was also estimated. Significant downregulation of EMX2OS and its negative correlation with miR-653-5p was observed in LUAD tissues. A significant relationship was revealed in EMX2OS with TNM stage, lymph node metastasis, and differentiation of LUAD patients, and associated with the poor prognosis of patients. EMX2OS suppressed the proliferation and metastasis of LUAD cells and negatively regulated the expression of miR-653-5p. The overexpression of miR-653-5p could reverse the inhibitory effect of EMX2OS on LUAD cells. In conclusion, EMX2OS served as a biomarker in LUAD that indicated patients’ prognosis and regulated cellular processes via regulating miR-653-5p.

## 1 Introduction

Non-small cell lung cancer (NSCLC) is the major pathological type of lung cancer together with small cell lung cancer, including lung adenocarcinoma (LUAD), large cell lung cancer, and lung squamous cell carcinoma. According to previous statistical data, NSCLC accounts for 85% of lung cancer, whereas LUAD accounts for about 60% [1,2]. LUAD has been considered the most common histological subtype of lung cancer. Surgical treatment, radiotherapy, chemotherapy, and target therapy are major methods for the clinical management of LUAD [3]. However, surgical treatment is only suitable for patients at an early to mild stage, and a huge number of patients are always diagnosed at a late stage and choose to receive radiotherapy or chemotherapy [4]. The radiotherapy and chemotherapy would induce gastrointestinal, myelosuppression, and other adverse reactions [5]. The unsatisfying survival of LUAD makes it necessary to explore novel therapeutic targets.

Non-coding RNAs (ncRNAs) occupy about 90% of human genomes, which have been illustrated to regulate various life processes, such as neurodevelopment, stem cell versatility, DNA damage, as well as tumor occurrence, and progression [6,7]. Among the various types of ncRNAs, the role of long non-coding RNAs (lncRNAs) in human diseases, especially the dysregulated lncRNAs, has gradually become a research hot spot. lncRNAs could serve as the competitive endogenous RNA (ceRNA) of microRNAs (miRNAs) co-regulating the development of malignant tumors, which were considered to possess great potential for serving as biomarkers. For instance, lncRNA JPX was demonstrated to modulate the onset and metastasis of lung cancer via the miR-33a-5p/Twist1 axis activating the Wnt/β-actin signaling pathway [8]. Meanwhile, some studies have been devoted to bioinformatics analysis to excavate potential biomarkers of lung cancer or other cancers. A recent lncRNA signature study identified a series of dysregulated lncRNAs in NSCLC, including EMX2 opposite strand/antisense RNA (EMX2OS) [9]. However, there was a lack of validation to confirm its specific significance and potential in serving as biomarkers in NSCLC or subtype cancers. Furthermore, the abnormal expression and functional role of EMX2OS were also gradually disclosed in various human cancers, including prostate cancer, ovarian cancer, and papillary thyroid cancer [10,11,12]. miR-653-5p was predicted to be a target of EMX2OS and regulates the progression of breast cancer, prostate cancer, and gastric cancer [12,13,14]. In NSCLC, miR-653-5p was also reported to mediate the effect of circular RNA circ-RAD23B on cell growth and invasion [15]. miR-653-5p might be a target of EMX2OS mediating the regulation of LUAD development by EMX2OS.

Therefore, this study focused on the expression and function of EMX2OS and miR-653-5p in LUAD, aiming to identify novel prognostic biomarkers and reveal the potential underlying molecular mechanism.

## 2 Methods

### 2.1 Study subjects

This study enrolled 117 patients diagnosed with LUAD and received surgical treatment in our hospital. None of the patients had received anticancer therapy before their diagnosis. Informed consent was obtained from patients and this study has been approved by the Ethics Committees of Qinhuangdao First Hospital. Paired tumor and normal tissues were collected and confirmed by at least two pathologists during surgery. The tissues were stored at −80°C for the following analyses.

### 2.2 Follow-up survey

To obtain the disease conditions of the patients after surgery, all enrolled patients were followed up through telephone or outpatient regular review. The follow-up time ranged from 3 to 60 months. The follow-up results were analyzed by Kaplan–Meier and Cox regression analysis.

### 2.3 Evaluation of EMX2OS and miR-653-5p expression

Tissues and cells were lysed with the Trizol reagent for the extraction of total RNA. The purity and concentration of the extracted RNA were evaluated with the Nanodrop 2000, and the ratio of OD260/280 ranged from 1.8 to 2.0 indicating a relatively high purity. cDNA was generated by the reverse transcription of extracted RNA with the PrimeScript RT Enzyme Mix I. The PCR was conducted with a TB Green Premix Ex Taq II kit on the real-time PCR system (Applied Biosystems, USA). The relative expression of EMX2OS (forward 5′-GTGACTTGCACAAGGACACAA-3′; reverse 5′-CCTGTCTGGCCATTCCTCT-3′) and miR-653-5p (forward 5′-GTGTTGAAACAATCTCTACTG-3′; reverse 5′-GAACATGTCTGCGTATCTC-3′) was calculated with the 2^−ΔΔct^ method with GAPDH (forward 5′-TATGATGATATCAAGAGGGTAGT-3′; reverse 5′-TGTATCCAAACTCATTGTCATAC-3′) and cel-miR-39 (5′-GATGAGGAGTGTCGTGGAGTCGGCAATTTCCTCATCCAAGCTG-3′) as the internal standard. The PCR conditions were as follows: 95°C for 5 min, 95°C denaturation for 30 s, 60°C annealing for 30 s, 72°C extensions for 30 s of 20 cycles, and 72°C for another 5 min

### 2.4 Cell culture

LUAD cells (A549, Calu-3, PC-9, and HCC827) and normal cells (MRC5) were obtained from ATCC and cultured in the 10% FBS-containing DMEM with 0.1% penicillin and streptomycin. Cells were incubated at 37°C with 5% CO_2_ until reaching 80% cell fusion and then cells were available for the following experiments.

### 2.5 Cell transfection

Cells were transfected with the EMX2OS plasmid to upregulate EMX2OS and with miR-653-5p mimic (5′-GUGUUGAAACAAUCUCUACUG-3′) or inhibitor (5′-CAGUAGAGAUUGUUUCAACAC-3′) for the regulation of miR-653-5p. The transfection was carried out with the employment of Lipofectamine 2000 (Invitrogen, USA) following the manufacturer’s instructions at room temperature. After 48 h of transfection, the expression of EMX2OS and miR-653-5p was detected to assess the transfection efficiency.

### 2.6 Cell counting kit-8 (CCK8) assay

Cells were seeded into 96-well plates and incubated for 0, 24, 48, and 72 h supplied with a completed culture medium followed by adding 10 μL of CCK8 to each well. After 2 h of CCK8 addition, OD450 of each well was measured with a microplate reader (Bio-Rad, USA).

### 2.7 Transwell assay

Cells (1 × 10^5^ cells per well) were seeded into the top chamber (coated with Matrigel for the invasion assay) of the 24-well Transwell plates and supplied with the FBS-free DMEM. The 10% FBS-containing medium was placed in the bottom chamber. The Transwell chambers were incubated for 24 h, and then the cells in the upper chambers were moved with the cotton swabs. The cells on the lower membrane surface were fixed and stained. The migrated and invasive cells were counted with a microscope in five random fields of each chamber.

### 2.8 Dual-luciferase reporter assay

EMX2OS wild-type and mutant-type vectors were established by cloning corresponding fragments into the pGL3 luciferase reporter vector (Promega, USA). The established vectors were co-transfected with a miR-653-5p mimic or an inhibitor into the LUAD cells with the help of Lipofectamine 2000 (Invitrogen, USA). The luciferase activity of EMX2OS was detected with the dual-luciferase reporter assay kit (Promega, USA) following the manufacturer’s instructions after 48 h of transfection and normalized to Renilla.

### 2.9 Statistical analysis

Data analysis was conducted with SPSS 26.0 and GraphPad Prism 7.0, and all data were expressed as mean ± standard deviation obtained from at least triple repeats. The association of EMX2OS with patients’ clinicopathological features was assessed by the *χ* ^2^ test. *P* < 0.05 indicated the statistical significance.

## 3 Results

### 3.1 Expression of EMX2OS and miR-653-5p in LUAD tissues

**Figure 1:**
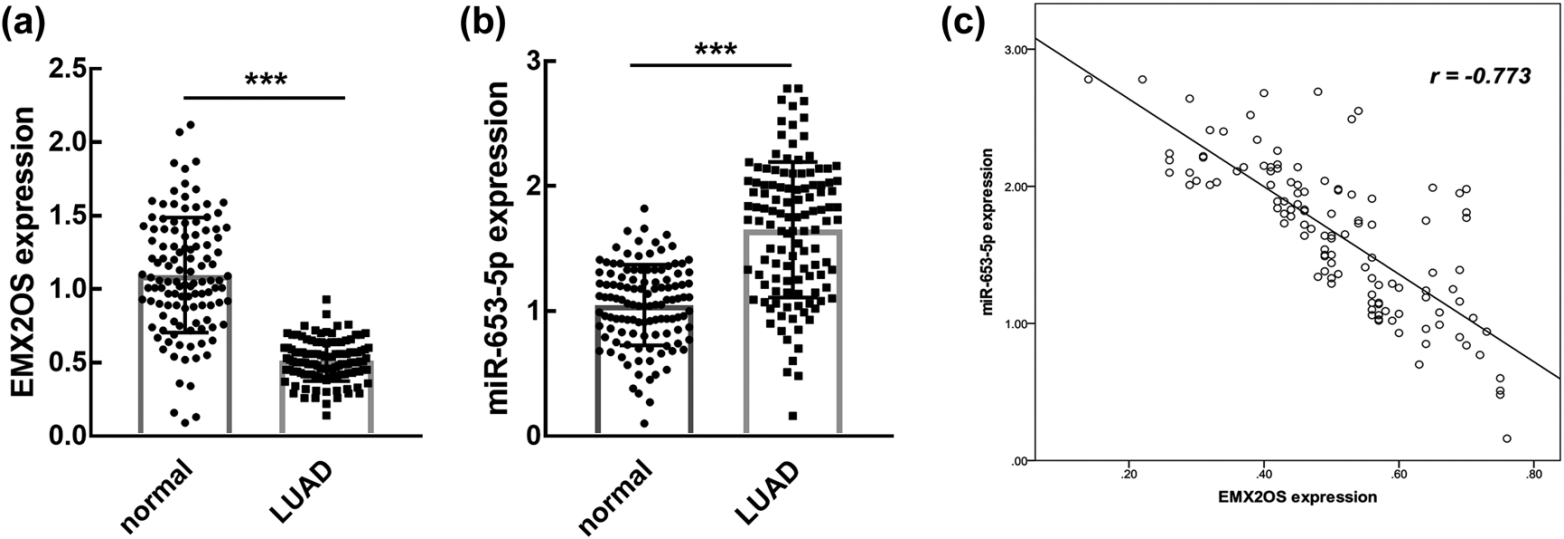
Expression of EMX2OS (a) and miR-653-5p (b) in LUAD tissues and their correlation evaluated by Spearman correlation analysis (c). Elevated EMX2OS in LUAD tissues was negatively correlated with the reduced miR-653-5p. *r* = −0.773. ***P < 0.001 compared with normal tissues.

In collected tissues, the significantly reduced EMX2OS level (Figure 1a) and the significantly elevated miR-653-5p level (Figure 1b) were observed in tumors compared with the corresponding normal tissues (*P* < 0.001). Additionally, reduced EMX2OS was negatively correlated with the increased miR-653-5p in tumor tissues (*r* = −0.773, *P* < 0.001, Figure 1c).

### 3.2 Association of EMX2OS with patients’ clinicopathological features and clinical prognosis

Patients were grouped into a low-EMX2OS group with 61 patients (39 males and 22 females) and a high-EMX2OS with 56 patients (40 males and 16 females) according to the average EMX2OS level in LUAD tumor tissues. Significant correlations were observed between EMX2OS levels and the TNM stage (*P* = 0.001), lymph node metastasis status (*P* = 0.020), and differentiation (*P* = 0.002) of LUAD patients (Table 1).

**Table 1:** Chi-square test evaluating the association of EMX2OS expression with patients’ clinicopathological features

	Cases (*n* = 117)	Low EMX2OS (*n* = 61)	High EMX2OS (*n* = 56)	*P*
Age				0.534
≤60	55	27	28	
>60	62	34	28	
Gender				0.387
Male	79	39	40	
Female	38	22	16	
Tumor size (mm)				0.177
≤30	53	24	29	
>30	64	37	27	
TNM stage				0.001
I–II	76	31	45	
III	41	30	11	
Lymph node metastasis				0.020
Yes	35	24	11	
No	82	37	45	
Differentiation				0.002
Well-moderate	73	30	43	
Poor	44	31	13	
Smoking				0.169
Yes	60	35	25	
No	57	26	31	

**Figure 2:**
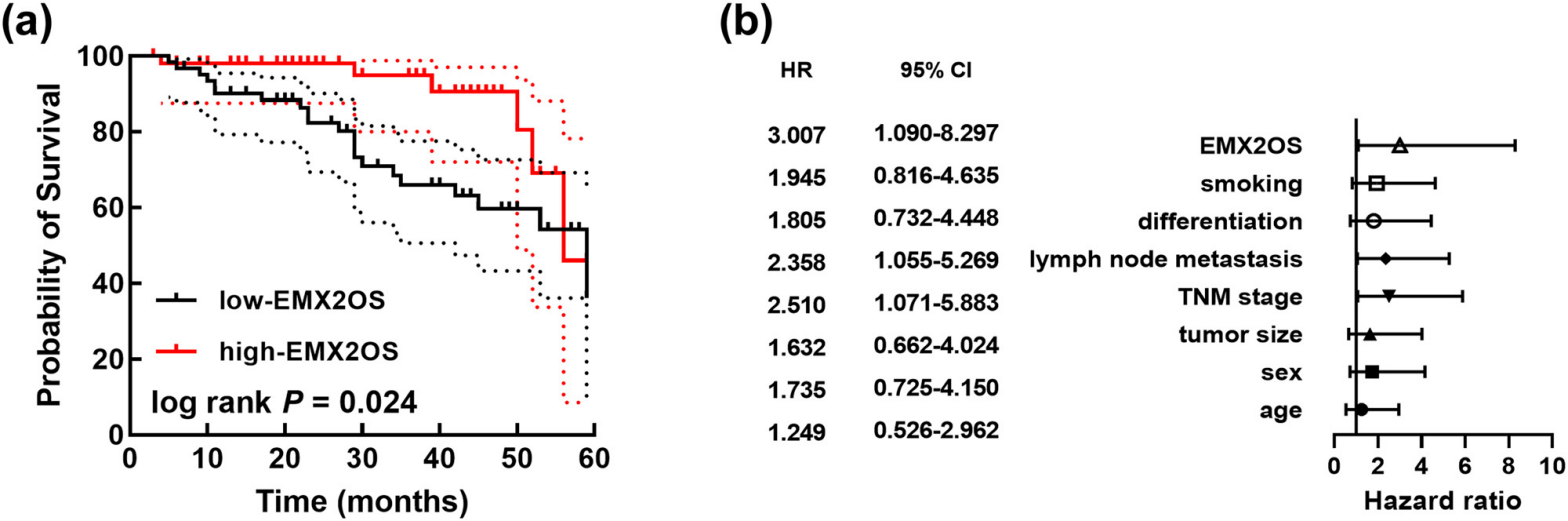
Kaplan–Meier followed by the log rank test assessed the overall survival rate of patients in 60 months (a). Cox regression analysis assessed the prognostic value of clinicopathological features and EMX2OS (b).

The follow-up data showed that the lower EMX2OS was significantly associated with patients’ lower survival rate (log rank *P* = 0.024, Figure 2a). Meanwhile, EMX2OS was identified as an independent prognostic factor (HR = 3.007, 95% CI = 1.090–8.297) together with the TNM stage (HR = 2.510, 95% CI = 1.071–5.883) and lymph node metastasis (HR = 2.358, 95% CI = 1.055–5.269, Figure 2b).

### 3.3 Regulatory effect of EMX2OS on miR-653-5p

**Figure 3:**
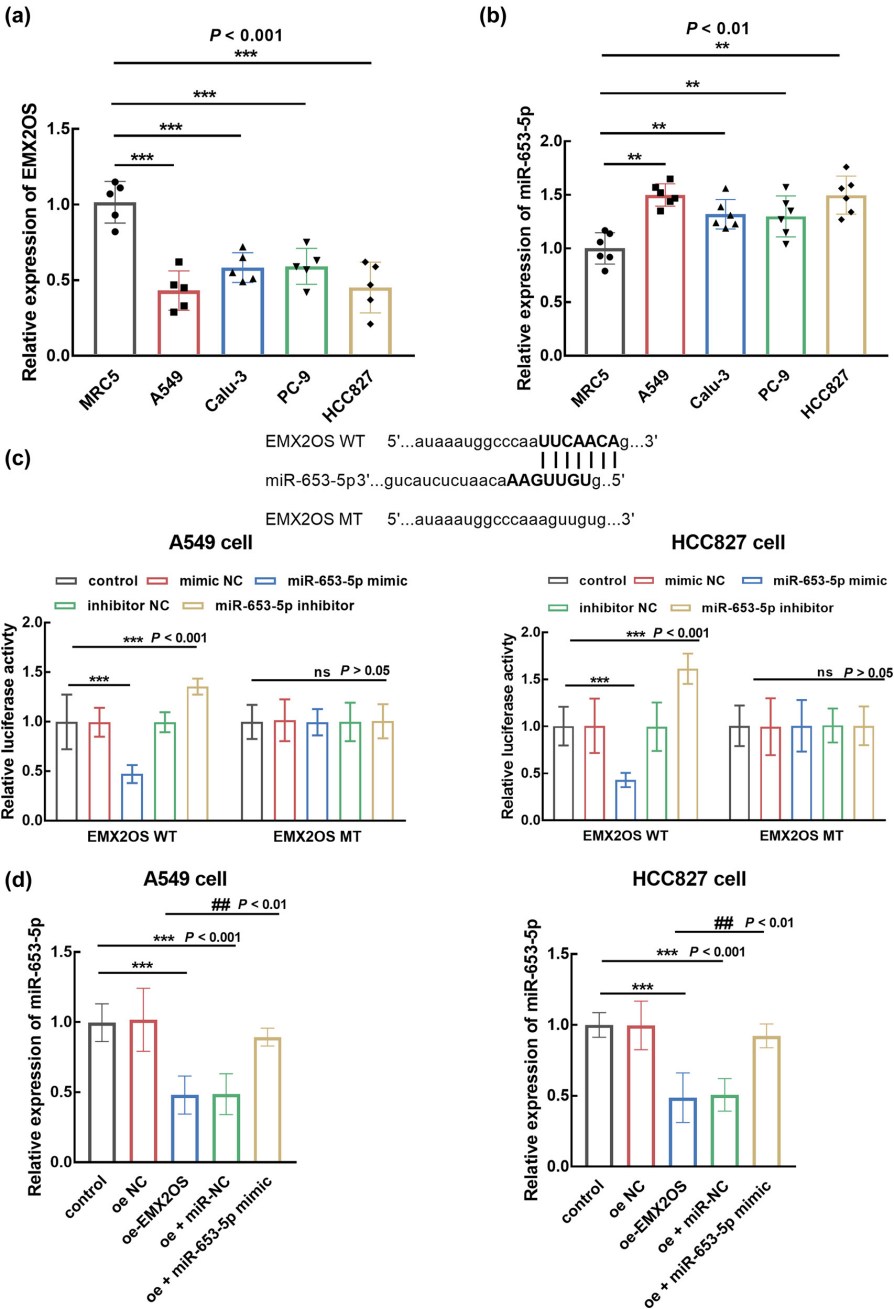
Expression of EMX2OS (a) and miR-653-5p (b) in LUAD cells. The regulatory effect of miR-653-5p on the luciferase activity of EMX2OS (c) and the effect of EMX2OS on miR-653-5p expression (d). EMX2OS negatively regulated the expression of miR-653-5p in LUAD cells, and the luciferase activity of EMX2OS was negatively regulated by miR-635-5p. **P < 0.01, ***P < 0.001, compared with normal cells or control group; ^##^ *P* < 0.01 compared with the oe-EMX2OS group.

Consistent with the dysregulation of EMX2OS and miR-653-5p in tumor tissues, the downregulation of EMX2OS (Figure 3a) and the upregulation of miR-653-5p (Figure 3b) were also found in the LUAD cells compared with normal cells (*P* < 0.01, *P* < 0.001). The interaction between EMX2OS and miR-653-5p was further assessed in A549 and HCC827 cells, which were sensitive to the dysregulation of EMX2OS and miR-653-5p. The overexpression of miR-653-5p dramatically suppressed the luciferase activity of EMX2OS, and significant enhancement was observed in the presence of miR-653-5p knockdown (*P* < 0.001, Figure 3c). Meanwhile, the overexpression of EMX2OS significantly suppressed the expression of miR-653-5p, which was reversed by the transfection of the miR-653-5p mimic (*P* < 0.01, *P* < 0.001, Figure 3d).

### 3.4 Effect of EMX2OS and miR-653-5p on the proliferation and metastasis of LUAD cells

**Figure 4:**
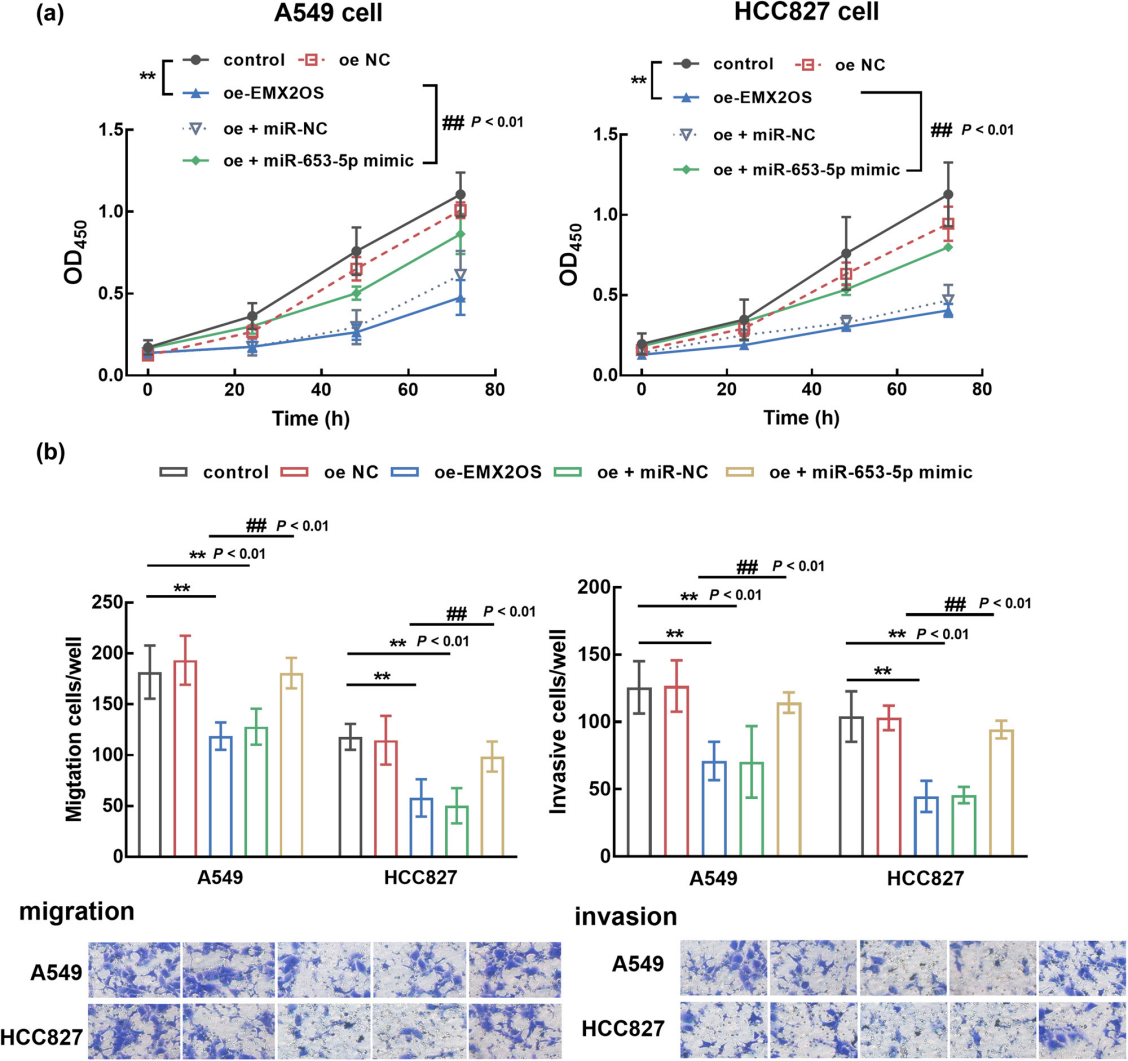
The function of EMX2OS and miR-653-5p in the proliferation (a) and metastasis (b) of LUAD cells. Overexpressing EMX2OS significantly suppressed cell growth and metastasis of LUAD cells, which was reversed by the overexpression of miR-653-5p. **P < 0.01, compared with the control group; ^##^ *P* < 0.01, compared with the oe-EMX2OS group.

In transfected cells, EMX2OS overexpression inhibited the proliferation of A549 and HCC827 cells compared with the untransfected cells (*P* < 0.01), while the inhibitory effect was attenuated by the elevated levels of miR-653-5p (*P* < 0.01, Figure 4a). In addition, the inhibitory effect of EMX2OS was also observed in the migration and invasion of A549 and HCC827 cells, which was also reversed by the overexpression of miR-653-5p (*P* < 0.01, Figure 4b).

## 4 Discussion

This study revealed that the reduced EMX2OS could indicate the adverse prognosis and malignant development of LUAD patients. Additionally, EMX2OS negatively regulates miR-653-5p, and miR-653-5p mediated the tumor inhibitory effect of EMX2OS on LUAD cells. As the major subtype of NSCLC, LUAD possessed a high incidence and mortality rate. Understanding the mechanism and developing a novel therapeutic target for LUAD have attracted special attention in the tumor research field. In this study, 117 LUAD patients were enrolled and decreased expression of EMX2OS was found in tumor versus normal tissues consistent with previous studies revealing its downregulation in lung cancer [9]. The dysregulation of EMX2OS was widely noted in a variety of cancer, such as gastric cancer, breast cancer, laryngeal cancer, and cervical cancer [13,14,16,17]. Previously, it was reported that EMX2OS predicted the recurrence-free survival of papillary thyroid cancer and correlated with the poor prognosis of kidney renal clear cell carcinoma [11,18]. Based on the previous reports about the function of EMX2OS in other cancers, the association of EMX2OS with clinical conditions was estimated. Alerted expression of EMX2OS was revealed to be related to LUAD development indicated by its close relationship with patients’ lymph node metastasis, poor differentiation, and advanced TNM stage [19,20]. Meanwhile, the poor overall survival of LUAD patients was related to the lower expression of EMX2OS, and multiple factors were identified as prognostic biomarkers of LUAD, including EMX2OS as well as lymph node metastasis and TNM stage.

EMX2OS was previously reported to be implicated in cellular processes correlated with disease development in various cancers. For example, Duan et al. demonstrated that EMX2OS modulated the proliferation, invasion, and sphere formation of ovarian cancer and therefore regulated tumor progression [10]. Through targeting miR-654-3p, EMX2OS suppressed cell growth and metastasis and induced cell apoptosis, which inhibited the progression of Wilms [21]. The inhibitory effect of EMX2OS was also displayed on the proliferation, migration, and invasion of LUAD cells. In mechanism, miR-653-5p was predicted to be a downstream ceRNA of EMX2OS, which was negatively correlated with EMX2OS and was regulated by EMX2OS dysregulation. More importantly, miR-653-5p was found to reverse the effect of EMX2OS on LUAD cells. miR-653-5p was illustrated to mediate the function of various lncRNAs and circRNAs. For instance, the promoted effect of lncRNA PRNCR1 on the proliferation, migration, and invasion of ovarian cancer was found to be alleviated by the miR-653-5p/ELF2 axis [22]. miR-653-5p has been suggested to mediate the promoted effect of circ-RAD23B on NSCLC cell growth and invasion [15]. Therefore, the inhibitory effect of EMX2OS on LUAD cells was speculated to be mediated by miR-653-5p.

Additionally, except for the exploration of a novel biomarker for LUAD, the present study confirmed some obvious advantages of ncRNAs in monitoring tumor progression. First of all, the significance of EMX2OS in prognosis prediction has been demonstrated in this study, and the potential molecular mechanism underlying the function of EMX2OS was disclosed, indicating the importance of lncRNAs in tumor-related research. Moreover, the expression of EMX2OS and miR-653-5p was detected in the tissues and cell lines by PCR, which is a relatively sensitive method. EMX2OS and miR-653-5p have also been detected in patients’ plasma samples [23,24]. The stable expression of ncRNAs in clinical samples could improve the efficiency and specificity of analysis methods.

However, some limitations need special attention in future investigations. This is a single-center study with a relatively small sample size, which might confine the significance of the clinical results. The source of lncRNAs and miRNAs, such as exosomes and other microparticles, is also a critical factor that might influence their function [25,26]. Hence, further studies should pay more attention to multiple center data with a larger sample size and focus on the difference in the function of ncRNAs with various sources. Moreover, the *in vitro* findings need further *in vivo* confirmation. Currently, animal models have been considered effective methods for *in vivo* investigation, especially for tumor progression [27,28]. Therefore, animal experiments are necessary for future investigations.

Taken together, downregulated EMX2OS in LUAD was identified as an independent prognostic indicator and associated with tumor progression. EMX2OS suppressed the proliferation and metastasis of LUAD cells via negatively modulating miR-653-5p.
